# Pembrolizumab-Induced Myasthenia Gravis With Myocarditis in the Setting of Metastatic Renal Cell Carcinoma

**DOI:** 10.7759/cureus.68318

**Published:** 2024-08-31

**Authors:** Phillip Taboada, Matthew Lee, Remington Hoyer, Zane Gray, Jue Wang

**Affiliations:** 1 School of Medicine, University of Texas Southwestern Medical School, Dallas, USA; 2 Department of Internal Medicine, University of Texas Southwestern Medical Center, Dallas, USA

**Keywords:** immune-related adverse events (iraes), renal cell carcinoma, myotoxicity, myasthenia gravis, immune checkpoint inhibitors

## Abstract

Immune checkpoint inhibitors (ICIs) like pembrolizumab are increasingly used for treating renal cell carcinoma (RCC), offering benefits such as enhanced specificity and activation of immunological memory. However, ICIs can lead to immune-related adverse events (irAEs), including rare but serious neurologic consequences such as myasthenia gravis (MG). We present a case of pembrolizumab-induced MG with concurrent orbital myositis and myocarditis.

A 69-year-old male with a history of pT3aN1 kidney cancer presented with abdominal pain, night sweats, and weight loss. Initial imaging revealed a retroperitoneal mass and a thyroid mass, and a biopsy confirmed papillary RCC. The patient began neoadjuvant therapy with pembrolizumab and axitinib. Three weeks post-initiation, he developed dysphagia, ptosis, and proptosis, which progressed with each pembrolizumab infusion. Hospitalization was required after the third cycle due to bilateral ptosis, heart block, and elevated troponins. Despite initial steroid treatment, symptoms persisted. Diagnoses of ICI-related MG (irMG) and myocarditis were established, and treatment included cessation of pembrolizumab, high-dose steroids, IVIGs, and a pacemaker for heart block. Post-discharge, the patient showed a slight improvement in ptosis but persistent dysphagia.

MG induced by ICIs is a rare but severe complication with rapid onset and progression, often presenting with bulbar involvement and a significant risk of respiratory failure. The therapeutic regimen for our patient, including high-dose methylprednisolone and IVIG, aligns with current recommendations. This case underscores the importance of recognizing cardiac irAEs like myocarditis in patients on ICIs, as early intervention can significantly affect outcomes. Despite therapeutic interventions, complete resolution of irMG symptoms is rare, and persistent sequelae are common.

This case highlights the critical need for vigilant monitoring and prompt management of neurologic and cardiac irAEs in patients undergoing ICI therapy. Clinicians should maintain a high index of suspicion for MG and myocarditis to improve diagnostic accuracy and patient outcomes.

## Introduction

Immune checkpoint inhibitors (ICIs), such as pembrolizumab, have gained traction in the treatment of renal cell carcinoma (RCC) due to their enhanced specificity in targeting neoplastic cells, activation of immunological memory, and potential for combination with other therapeutic modalities [1,2]. However, the therapeutic benefits of ICIs are counterbalanced by the risk of immune-related adverse events (irAEs), which, although infrequent, can be severe.

Neurologic irAEs, such as myasthenia gravis (MG), autoimmune encephalitis, and central demyelinating disorders, are documented but rare, with an incidence ranging from 1% to 12% [3,4]. These neurologic complications can vary in severity and, in some cases, may be fatal [5]. The pathogenesis of ICI-induced neurotoxicity is attributed to the inhibition of CTLA-4 and PD-1 pathways, leading to unrestrained T-cell activation. This prolonged activation results in the release of proinflammatory cytokines and chemokines, which induce endothelial cell activation and disrupt the integrity of the blood-brain barrier [1]. ICI-related MG (irMG) can present concomitantly with other irAEs, particularly myositis and sensorimotor polyneuropathy, complicating the clinical presentation and management [3]. The overlapping symptoms pose a diagnostic challenge and necessitate a high index of suspicion in patients undergoing ICI therapy.

In this case report, we describe a patient who developed pembrolizumab-induced MG with concurrent orbital myositis and myocarditis. The patient exhibited an initial limited response to high-dose corticosteroid therapy but subsequently demonstrated clinical improvement and stabilization.

## Case presentation

**Figure 1:**
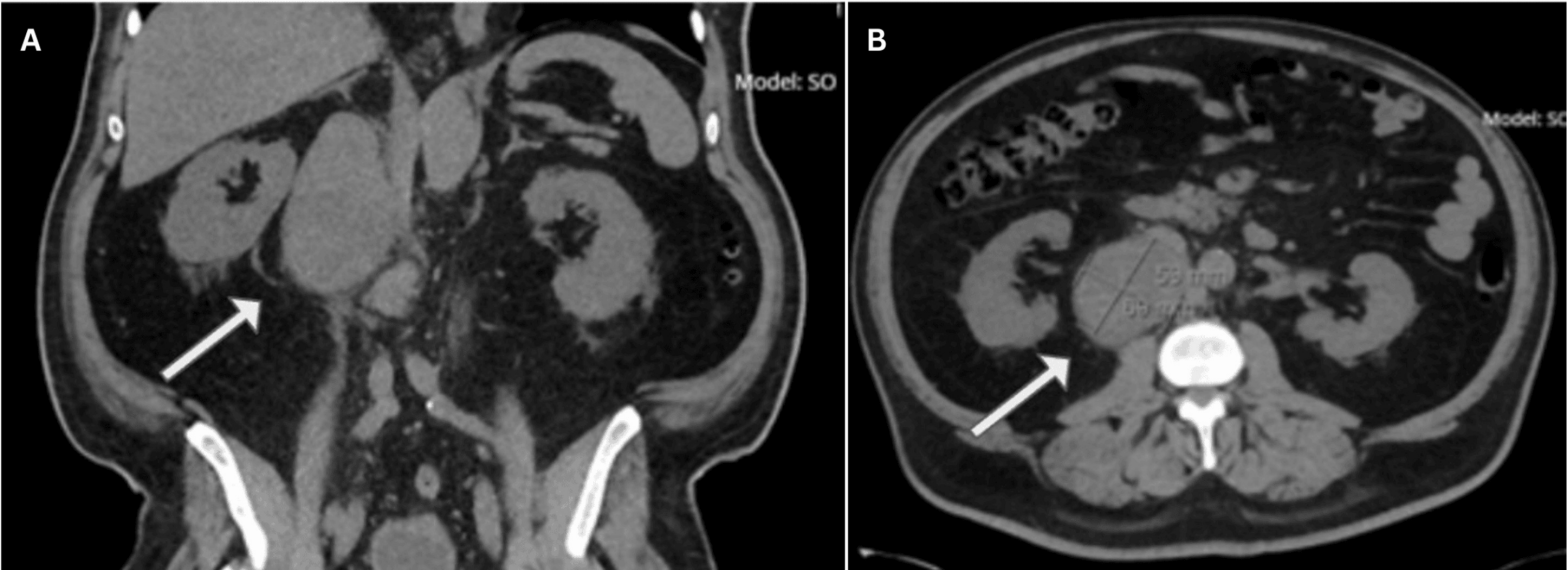
(A, B) CT-guided core needle biopsy confirmed the retroperitoneal mass as RCC (white arrows) CT: computed tomography, RCC: renal cell carcinoma

**Figure 2:**
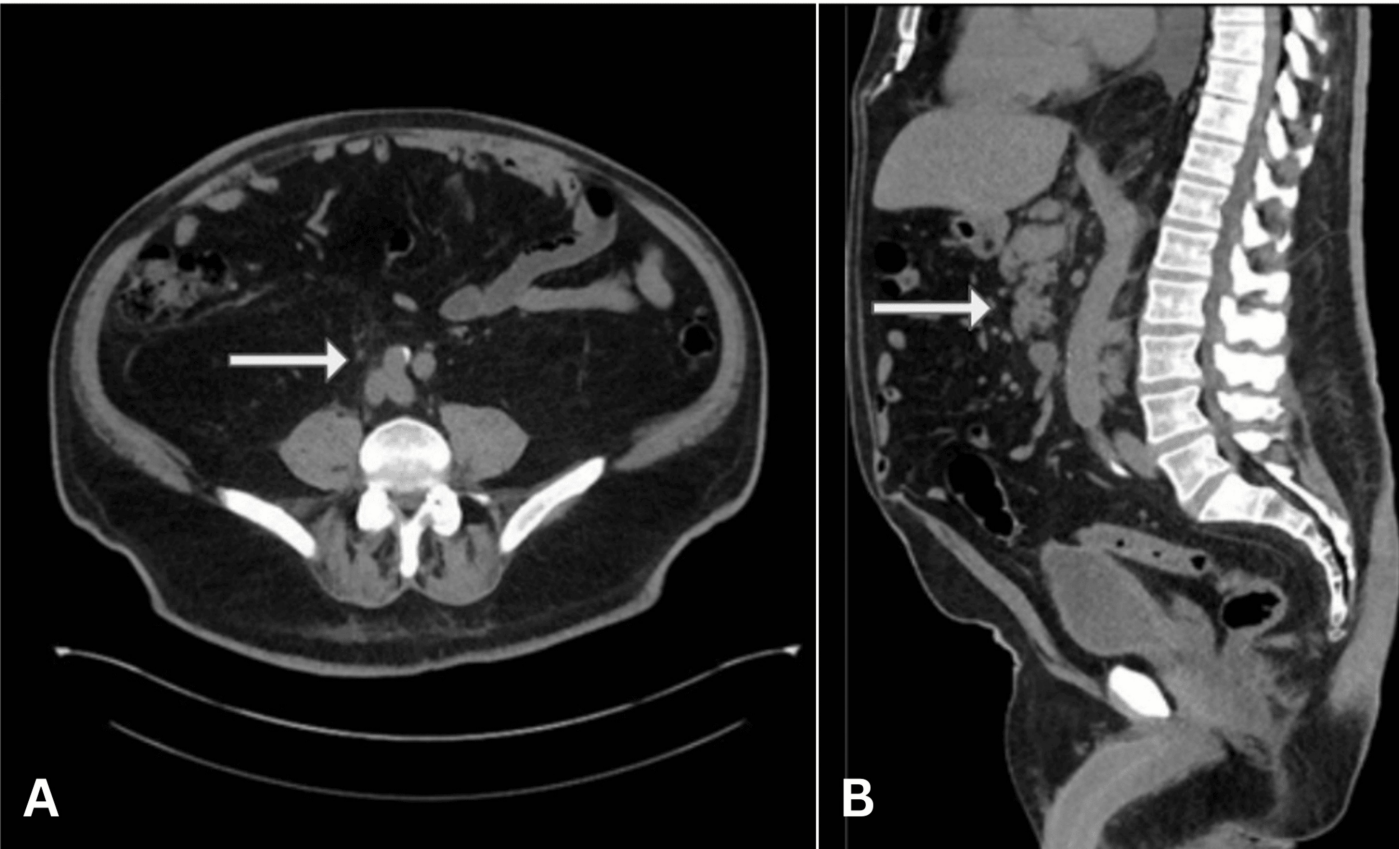
(A, B) Pathologically enlarged right retroperitoneal lymph nodes directly abutting the distal inferior vena cava (white arrows)

This case concerns a 69-year-old male patient with a past medical history of pT3aN1 kidney cancer (Figure 1) who presented to the emergency room with a three-week history of constant pain radiating from the abdomen to the groin on the right side, night sweats, and 15 lb. (6.8 kg) weight loss in recent months. This led to CT imaging, which revealed a large retroperitoneal mass, 3.8 x 3.5 cm in size, as seen in Figure 2, alongside a thyroid mass seen on ultrasound. A CT-guided core needle biopsy confirmed the retroperitoneal mass as a papillary RCC. CT without IV contrast was done to further evaluate the thyroid mass and showed an enlarged right thyroid lobule with bilateral nodules, measuring up to 2 cm in diameter. Thyroid labs were obtained and showed a euthyroid process with detectable but subnormal thyroid-stimulating hormone values and negative thyroid peroxidase enzymes, suggesting an illness of nonthyroidal origin.

The patient was started on 200 mg IV pembrolizumab every 21 days and 5 mg oral axitinib every 12 hours as neoadjuvant therapy. Dysphagia and right upper eyelid ptosis and proptosis began gradually three weeks after the first infusion of pembrolizumab. These symptoms worsened throughout the day and did not improve with rest. Each subsequent infusion increased his symptom severity, eventually resulting in bilateral ptosis and proptosis after the third treatment cycle (day 63 after initiation of pembrolizumab treatment). The patient was administered a steroid after his third infusion without improvement in his symptoms. An ice pack test resulted in a modest improvement of ptosis. He was hospitalized for further workup at this point.

In addition to his ocular symptoms, he was admitted for heart block with a heart rate in the 20s and elevated troponins. A heart catheterization performed at that time revealed no blockages or myocardial infarction, and no stents were placed. A pacemaker was implanted during the same admission, but there was little clinical suspicion of a relationship between his heart block and the recent initiation of pembrolizumab. A transthoracic echocardiogram conducted in October 2023 was normal. During the admission, he received a single dose of IV methylprednisolone for his eye symptoms, which provided limited benefit.

In the hospital, the patient did not exhibit neck, jaw, extremity weakness, or respiratory distress. He did, however, report some orthopnea along with aphonia, dysphagia to liquid, and generalized fatigue. He was found to have extraocular muscle abnormality, facial weakness, tongue atrophy, an absent gag reflex, and dysarthria by neurology. Creatine phosphokinase (CPK) was elevated at 566 mcg/L (normal: 10-120 mcg/L), erythrocyte sedimentation rate 31 mm/hr (normal: <20 mm/hr), C-reactive protein 12.8 mg/L (normal: 8-10 mg/L), and aldolase 13 U/L (normal: 1.0-7.5 U/L). As shown in Table 1, a panel of antibodies commonly involved in MG was unremarkable aside from a positive anti-LRP4 value. The patient did not tolerate an MRI of the brain and orbits due to dysphagia when supine.

**Table 1:** Patient test results for common antibodies involved in MG MG: myasthenia gravis

Antibody	Reference range	Value
Muscle-specific receptor tyrosine kinase Ab	0.00-0.02 nmol/L	0.00 nmol/L
Striated muscle Ab, IgG screen	<1:40	<1:40
Acetylcholine-modulating Ab	≤45%	12
P/Q-type calcium channel Ab	≤0.02 nmol/L	0.00 nmol/L
anti-LRP4	Negative	Positive

The diagnosis of irMG was clinically established due to the close period from initiation of pembrolizumab and the lack of typical antibody findings seen in idiopathic MG patients. Furthermore, a probable causal relationship is supported by a score of 7 on the Naranjo Adverse Drug Reaction Probability Scale [6].

Treatment in the hospital was initiated with prompt cessation of pembrolizumab and 1 g IV methylprednisolone daily for two days, followed by oral prednisone taper alongside IVIG (2 g/kg divided into 0.4 g/kg doses) over five days. His ptosis improved slightly with IVIG treatment, with more pronounced improvements observed on the right side. CPK normalized to 101 mcg/L shortly after initiation of this steroid regimen. The patient was discharged on a steroid taper of 60 mg for three days, followed by 50 mg for seven days and 40 mg for 30 days, and pyridostigmine 30 mg three times daily.

Several weeks after being discharged, the patient presented to the oncology clinic due to the inability to open his right eyelid and persistent dysphagia to liquids. The persistence of symptoms supported a designation of grade 4 toxicity from ICI therapy [7].

## Discussion

MG represents a rare yet potentially life-threatening complication following ICI therapy, characterized by acute onset and rapid progression. The median onset time of irMG typically occurs within one month of initiating ICI treatment, with ptosis being the most frequently reported initial symptom. This is significant because ptosis and diplopia are uncommon in non-irMG forms of myositis. This finding may indicate concomitant neuromuscular junction dysfunction, which may further complicate the delineation of presenting symptoms and their causes. Checking for improvement of ptosis with an ice pack applied to the fatiguable eyelid for two minutes is an inexpensive test that can support a more complete MG workup.

Notably, irMG often presents with bulbar involvement, leading to respiratory failure within a week in up to 50% of patients, necessitating invasive ventilation. Despite therapeutic interventions, complete resolution of irMG symptoms and deficits is rare, resulting in persistent sequelae of the disease process. Lyon et al. [8] reported a mortality rate of 30% associated with ICI-mediated MG, underscoring the serious nature of this complication. Careful history-taking and heightened observation for the development of muscle weakness are imperative in the early detection of MG and other neuropathies associated with ICI treatment.

In both immune checkpoint irMG and conventional MG, recommended therapeutic approaches include plasmapheresis, IVIG, high-dose steroids for acute symptomatic management, and pyridostigmine, which also demonstrates clinical efficacy. Success has been achieved with high-dose methylprednisolone (1 g) as the primary treatment modality, with plasmapheresis or long-term maintenance on steroid-sparing agents such as azathioprine and mycophenolate mofetil being recommended in cases where corticosteroid therapy fails. Conversely, some experts advocate for initiating IVIG and plasmapheresis regardless of MG severity, favoring these interventions over steroids and other agents due to their rapid immunosuppressive effects. Additional therapeutic agents utilized in the management of irMG and MG include rituximab, antitumor necrosis factor alpha (TNF-α) agents, and cyclosporin. The current regimen of 1 g IV methylprednisolone daily for two days, followed by an oral prednisone taper alongside IVIG (2 g/kg divided into 0.4 g/kg doses over five days), is in line with literature recommendations for treating myositis and neurologic irAEs induced by pembrolizumab. This approach aligns with guidelines to rapidly suppress the immune response, reduce inflammation, and manage severe irAEs effectively.

It has been hypothesized that among patients who survive irMG or neurotoxicity related to irAEs, there may be a tendency toward more favorable outcomes. This proposition suggests that such outcomes could potentially be linked to heightened tumor responsiveness, although this mechanism remains speculative. While some studies have indicated that the development of irAEs might be associated with therapeutic responses in certain clinical contexts involving ICIs, it's important to note that this relationship is not fully established, particularly in the case of irMG. Further research is needed to elucidate the complex interplay between irAEs, tumor response, and patient outcomes in the context of immunotherapy.

This patient's case further underscores the critical importance of evaluating cardiac symptoms in the context of immunomodulatory therapy. Pembrolizumab, an ICI, is associated with irAEs, including myocarditis, which, although rare, can be life-threatening. The incidence of pembrolizumab-induced myocarditis is estimated to be between 0.06% and 1.14%, with a high mortality rate if not promptly diagnosed and treated [9,10].

In this patient, the development of heart block and elevated troponins suggested myocardial involvement. However, these cardiac symptoms were initially not connected to his immunotherapy regimen, leading to a missed diagnosis of myocarditis. ICI-induced myocarditis can present with a wide range of symptoms, from asymptomatic elevations in cardiac biomarkers to life-threatening arrhythmias and heart failure [11]. The diagnosis is challenging due to its nonspecific presentation and symptom overlap with other cardiovascular conditions. Had the connection to his immunomodulation been made at an appropriate time, he might not have needed a pacemaker placed for his heart block.

Current guidelines recommend maintaining a high index of suspicion for myocarditis in patients receiving ICIs who present with new-onset cardiac symptoms, even if routine cardiac imaging and catheterization do not reveal typical ischemic changes [12]. Early recognition and intervention with high-dose corticosteroids or other immunosuppressive therapies are crucial for managing these patients and improving outcomes [13].

## Conclusions

This case highlights the necessity of integrating clinical findings with the patient’s treatment history to ensure prompt diagnosis and management of potential irAEs. Clinicians should be vigilant for signs of myocarditis in patients undergoing immunomodulatory therapy and consider it in the differential diagnosis when cardiac symptoms arise, even in the absence of traditional risk factors or evident ischemic disease. This approach can lead to the timely initiation of appropriate therapies, potentially reducing the morbidity and mortality associated with these adverse events.
